# Expression of *FOXP3*, *CD14*, and *ARG1* in Neuroblastoma Tumor Tissue from High-Risk Patients Predicts Event-Free and Overall Survival

**DOI:** 10.1155/2015/347867

**Published:** 2015-06-16

**Authors:** Sara Stigliani, Michela Croce, Fabio Morandi, Paola Scaruffi, Valentina Rigo, Barbara Carlini, Carla Manzitti, Anna Rita Gigliotti, Gian Paolo Tonini, Vito Pistoia, Silvano Ferrini, Maria Valeria Corrias

**Affiliations:** ^1^Physiopathology of Human Reproduction, IRCCS A.O.U. San Martino-IST, 16132 Genoa, Italy; ^2^Laboratory of Biotherapy, IRCCS A.O.U. San Martino-IST, 16132 Genoa, Italy; ^3^Laboratory of Oncology, IRCCS Istituto Giannina Gaslini, 16148 Genoa, Italy; ^4^Oncology Unit, IRCCS Istituto Giannina Gaslini, 16148 Genoa, Italy; ^5^Epidemiology, Biostatistics and Committees Unit, IRCCS Istituto Giannina Gaslini, 16148 Genoa, Italy; ^6^Neuroblastoma Laboratory, Pediatric Research Institute, Fondazione Città della Speranza, 35127 Padua, Italy

## Abstract

The prognosis of children with metastatic neuroblastoma (NB) > 18 months at diagnosis is dismal. Since the immune status of the tumor microenvironment could play a role in the history of disease, we evaluated the expression of *CD45*, *CD14*, *ARG1*, *CD163*, *CD4*, *FOXP3*, *Perforin-1* (*PRF1*), *Granzyme B (GRMB)*, and *IL-10* mRNAs in primary tumors at diagnosis from children with metastatic NB and tested whether the transcript levels are significantly associated to event-free and overall survival (EFS and OS, resp.). Children with high expression of *CD14*, *ARG1* and *FOXP3* mRNA in their primary tumors had significantly better EFS. Elevated expression of *CD14*, and *FOXP3* mRNA was significantly associated to better OS. *CD14* mRNA expression levels significantly correlated to all markers, with the exception of *CD4*. Strong positive correlations were found between *PRF1* and *CD163*, as well as between *PFR1* and *FOXP3*. It is worth noting that the combination of high levels of *CD14*, *FOXP3*, and *ARG1* mRNAs identified a small group of patients with excellent EFS and OS, whereas low levels of *CD14* were sufficient to identify patients with dismal survival. Thus, the immune status of the primary tumors of high-risk NB patients may influence the natural history of this pediatric cancer.

## 1. Introduction

Neuroblastoma (NB) is a pediatric neuroectodermal solid tumor with a heterogeneous clinical behavior [1]. Despite intensive multimodal therapy, patients presenting with metastatic disease, that is, stage 4 according to INSS [2] or stage M according to INRG-SS [3], aged more than 18 months at diagnosis have dismal survival rate. The search for powerful prognostic markers for this subset of patients is aimed to identify the cases that can be cured by standard therapy and the ultra-high-risk cases that need to be enrolled in new experimental trials.

Recently, the presence of high levels of NB-related molecular markers in bone marrow (BM) and peripheral blood (PB) samples at diagnosis has been shown to be highly predictive of event free survival (EFS) and overall survival (OS) [4]. However, a prognostic marker in the primary tumor could be helpful to improve patients' stratification. Unfortunately, although several gene expressions profiling studies of primary tumor specimens have identified prognostic signatures [5–12], so far none of the latter has a predictive power within the subset of stage 4 patients aged > 18 months at diagnosis.

It is increasingly evident that the tumor microenvironment plays an important role in driving the fate of antitumor response (see [13] for a review). Indeed, several membrane-bound or soluble factors produced by normal and neoplastic cells in the tumor microenvironment may downregulate the antitumor immune response and greatly influence the natural history of cancer. Recently, specific gene signatures related to a successful immune response and to tumor rejection processes have been related to tumor outcome in different tumors [14].

In human NB, information on the presence and activity of specific subsets of immune suppressive cells and soluble factors in the primary tumors is scanty [15]. Facchetti et al. [16] showed that NB primary tumors show different degrees of lymphocyte infiltration, but no correlation with survival was found. A gene expression study performed on primary tumors [12] suggested a negative role for myeloid-derived suppressor cells in the prognosis of metastatic NB patients. Recently, the same authors [17, 18] demonstrated that the inclusion of 5 inflammation related genes increased the predictive power of the gene signature, since tumors from high-risk NB patients present a greater infiltration of CD163+, M2-type, tumor associated macrophages (TAMs). Interestingly, NKT cells can be instructed to selectively kill these TAMs [18].

An important immune suppressive role is ascribed to CD4+CD25^high^FoxP3+ T cells, also termed Treg cells. No difference in their number was found in PB samples from a small cohort of low- and high-risk patients [19]. However, Tilak et al. have recently shown that the frequency of Treg in PB samples was higher in NB patients than in healthy children and that frequency was reduced after chemotherapy [20]. Nevertheless, the analysis of Treg in several tumor types indicated that FoxP3+ expression may be related to CD4+ T-cell activation rather than to an immune suppressive phenotype [21, 22]. This finding has important clinical implications since depletion of CD4+CD25^high^FoxP3+ cells has been proposed as a tool to enhance tumor responses. Indeed, Carlson and coworkers [23] recently demonstrated that NB tumor-infiltrating CD4+ T cells can be activated in the tumor milieu, but not in the periphery. Gowda et al. [19] surprisingly found that the immunosuppressive cytokine IL-10, produced by Treg, T regulatory type 1 (Tr1) cells [24], and cells of the innate immunity, such as NK and macrophages, was elevated in PB of patients with low-risk NB, suggesting a protective role of innate immunity.

To gain insight into the role of different immune cell populations in the natural history of metastatic NB, we have evaluated the mRNA expression of the following molecular markers in 41 primary tumors at diagnosis:* CD45*: all leukocytes,* CD14*: monocyte-macrophages,* ARG1*: activated macrophages,* CD163*: M2 TAM,* CD4*: T helper cells,* FOXP3*: Treg,* Perforin-1 (PRF1)*, and* Granzyme B (GRMB)*: cytotoxic T lymphocytes and activated NK cells. In addition we evaluated the mRNA expression of the immune suppressive cytokine* IL-10*. We then tested whether expression of these genes significantly correlated to survival.

## 2. Materials and Methods

### 2.1. Patients and Tumors

Patients included in the study were diagnosed in Italy with stage 4 NB between December 1992 and October 2006. Disease staging [2] was made at the referring oncology center and centrally reviewed at the Gaslini Institute. The median age at diagnosis was 3.4 years (range 1.5–6.3) and the median follow-up was 46.7 months (range 0.3–172.4). Patients were treated according to protocols NB-92, NB-95, and NB-97, which include induction therapy followed by myeloablative chemotherapy with autologous stem cell transplantation for consolidation. Survival rates for these protocols have been demonstrated to be similar [25] and are in line with the expected survival rate for patients with metastatic NB aged > 18 months at diagnosis [3]. Follow-up data at January 2014 were retrieved from the Italian Neuroblastoma Registry (INBR) [25]. Patients' characteristics are reported in Table 1.

After histological diagnosis, an aliquot of the primary tumor surgically resected at diagnosis was centralized at the Gaslini Institute and stored at −80°C until RNA was extracted. Only tumors with neoplastic cell content higher than 80% were included.

The study was approved by the Institutions' Ethical Committees and all analyses were performed according to the Helsinki declaration.

### 2.2. RNA Extraction and RT-qPCR Analysis

Total RNA was extracted from primary tumors as previously described [26]. One hundred ng of total RNA was reverse transcribed and then amplified for each molecular marker in duplicate by qPCR, using the following assays from Life Technology (Life Technologies Europe BV, Monza, Italy):* CD45*: Hs00365634_g1,* CD14*: Hs02621496_s1,* CD163*: Hs00174705_m1,* ARG1*: Hs00968979_m1,* CD4*: Hs01058407_m1,* FOXP3*: Hs00203958_m1,* IL10*: Hs00961622_m1,* PRF1*: Hs00169473_m1,* GZMB*: Hs01554355_m1, and primers and probe for *β*2-microglobulin (*B2M*) [27]. The level of expression of each marker was normalized to the expression of* B2M*, according to the delta Ct method [28] and results were reported as 2^−delta Ct^. All markers were tested on a panel of 10 NB cell lines and none of these markers was expressed by the tumor cells themselves. Moreover, to exclude DNA contamination the cDNA obtained in the absence of reverse transcriptase was included in each qPCR assay. Water was also run as negative control.

### 2.3. Statistical Analysis

The Wilcoxon-Mann-Whitney test was used to compare median values, and the Spearman *ρ* coefficient was used to assess correlation between variables. Event-free and overall survival (EFS and OS, resp.) analyses were performed according to the Kaplan-Meier method and compared by the log-rank test. A *P* value < 0.05 was considered as statistically significant. Analyses were made using the Prism software (GraphPad Software Inc., La Jolla, CA).

## 3. Results

### 3.1. Expression of Molecular Markers for Different Immune Cell Populations in Primary NB Tumors

**Figure 1:**
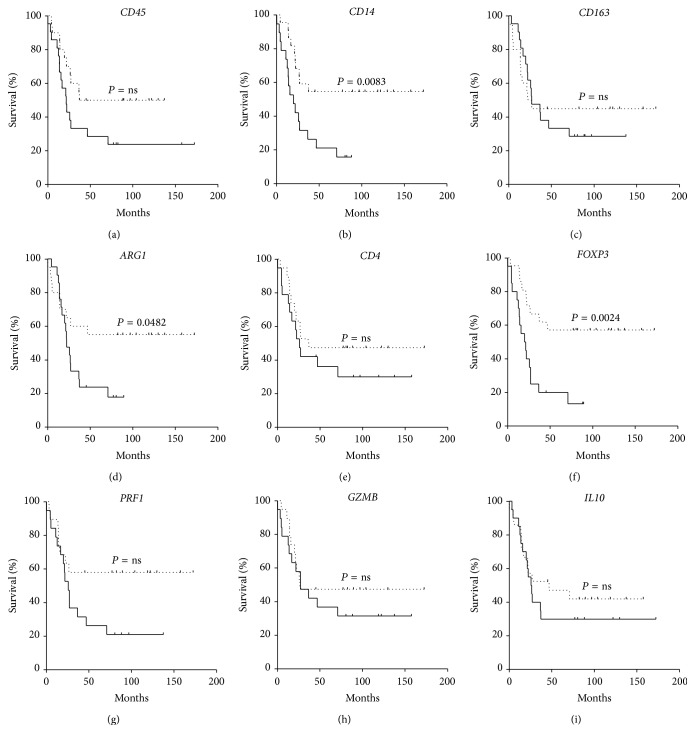
Kaplan-Meyer plots of EFS of stage 4 patients stratified according to the level of mRNA expression below (continuous line) or above (dotted line) the median of* CD45* (a),* CD14* (b),* CD163* (c),* ARG1* (d),* CD4* (e),* FOXP3* (f),* PRF1* (g),* GZMB* (h), and* IL10* (i).

We evaluated the expression of* CD45*,* CD14*,* CD163*,* ARG1*,* CD4*,* FOXP3*,* PRF1*,* GMZB*, and* IL10* mRNAs in primary tumor samples taken at diagnosis from 41 patients with metastatic NB >18 months at diagnosis. After normalization to* B2M* expression, the median expression value was used to stratify patients and correlation with EFS and OS was tested by the log rank test. High-risk patients with high expression of* CD14*,* ARG1*, and* FOXP3* mRNA in their primary tumors had a significantly better EFS (Figures 1(b), 1(d), and 1(f), *P* = 0.0083, *P* = 0.0482, and *P* = 0.0024, resp.). In addition, a trend toward a better survival was seen for patients with high expression of* CD45* and* PRF1* (Figures 1(a) and 1(g), resp.).

**Figure 2:**
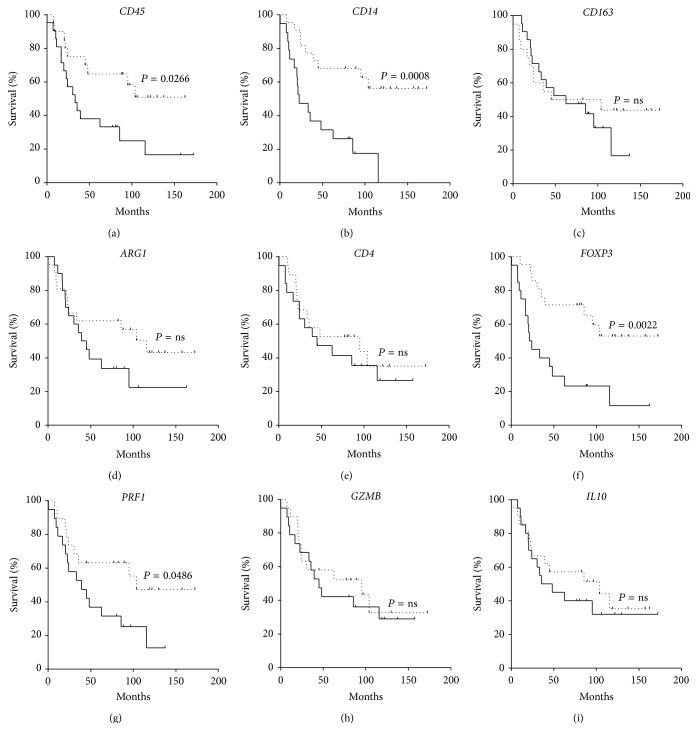
Kaplan-Meyer plots of OS of stage 4 patients stratified according to the level of mRNA expression below (continuous line) or above (dotted line) the median of* CD45* (a),* CD14* (b),* CD163* (c),* ARG1* (d),* CD4* (e),* FOXP3* (f),* PRF1* (g),* GZMB* (h), and* IL10* (i).

The OS was significantly better for patients with high* CD14* and* FOXP3* RNA expression in their primary tumors at diagnosis (Figures 2(b) and 2(f), *P* = 0.0008 and *P* = 0.0022, resp.). It is worth noting that all the patients with* CD14* expression below the median died of disease. Overall survival was also better for patients that had high* CD45* and* PRF1* expression (Figures 2(a) and 2(g), *P* = 0.0266 and *P* = 0.0486, resp.).

Levels of* CD163*,* CD4*,* GZMB*, and* IL10* mRNA in primary tumors of stage 4 NB patients were never associated to different EFS or OS (Figures 1 and 2, resp.).

### 3.2. Correlation of Expression Levels of Molecular Markers

**Figure 3:**
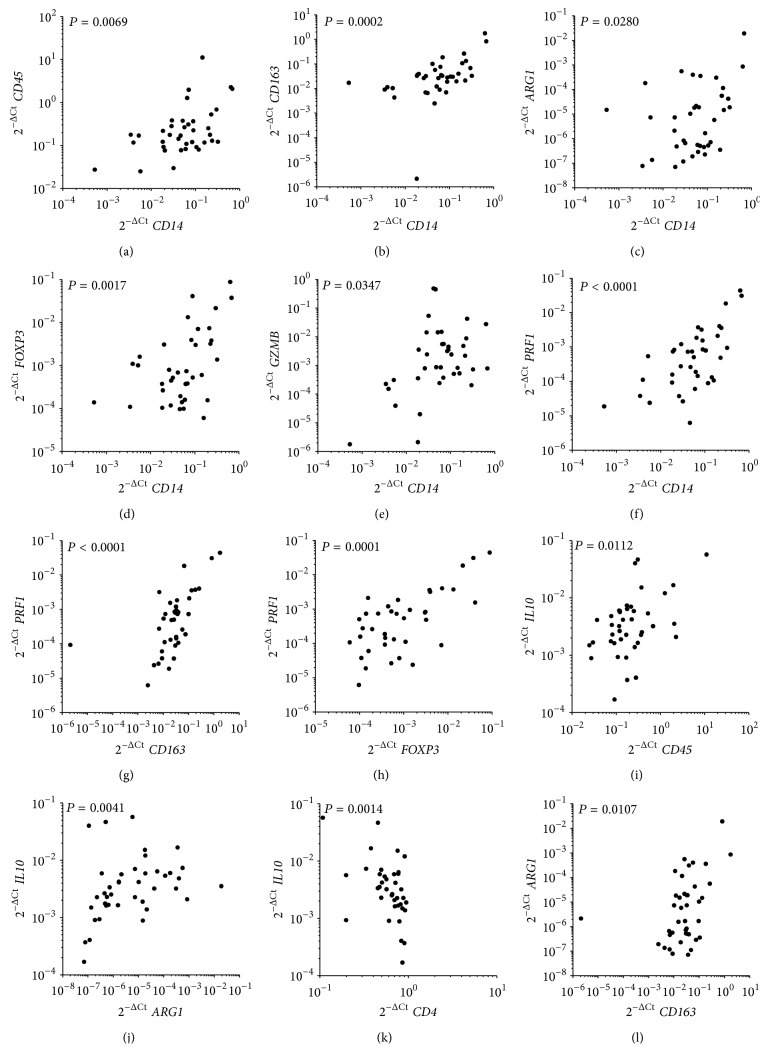
Significant *r* Spearman's correlations between* CD14* and* CD45* (a),* CD163* (b),* ARG1* (c),* FOXP3* (d),* GZMB* (e), and* PRF1* (f); between* PRF1* and* CD163* (g) and* FOXP3* (h); between* IL10* and* CD45* (i),* ARG1* (j), and* CD4* (k); and between* CD163* and* ARG1* (l).

We then analyzed potential correlation in the expression of the molecular markers.* CD14* mRNA expression levels significantly correlated to all the other markers (Figures 3(a) to 3(f)), with the exception of* CD4* (not shown). Surprisingly, strong positive correlations were found between* PRF1* and* CD163*, as well as between* PFR1* and* FOXP3* (Figures 3(g) and 3(h), resp.). Positive correlations were also found between* IL10* and* ARG1*, between* IL10* and* CD4* (Figures 3(j) and 3(k), resp.), and between* CD163* and* ARG1* (Figure 3(l)).

### 3.3. Predictive Power of Molecular Markers

**Figure 4:**
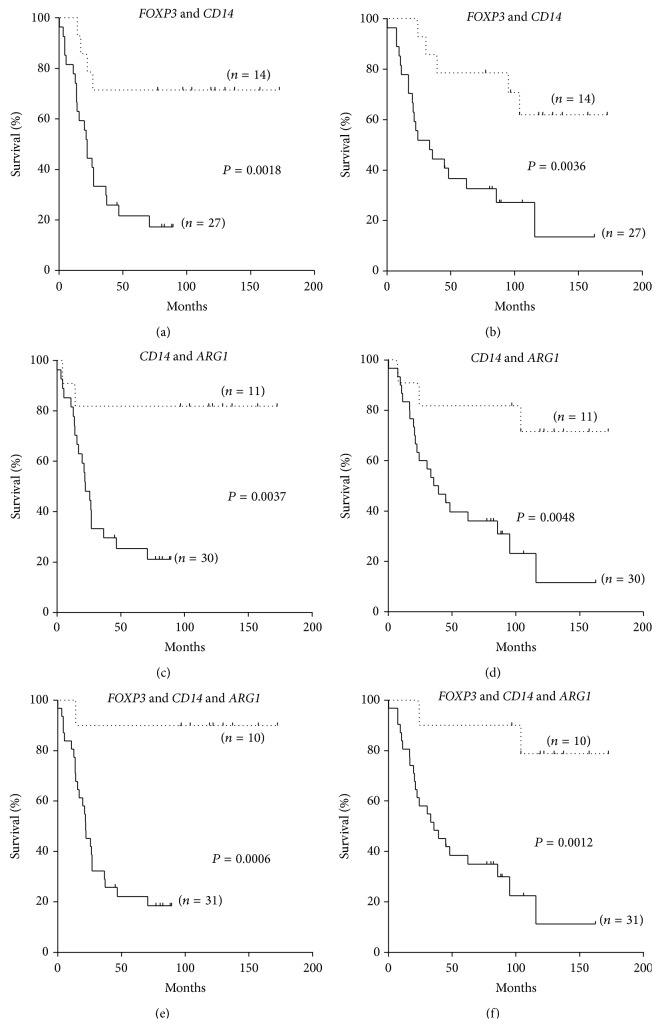
Kaplan-Meyer plots of EFS (left panels) and OS (right panels) of stage 4 patients stratified according to the level of mRNA expression below (continuous line) or above (dotted line) the median for* FOXP3* and* CD14* ((a) and (b)),* CD14* and* ARG1* ((c) and (d)), and for* FOXP3*,* CD14*, and* ARG1* ((e) and (f)). The number of patients in each curve is given in brackets.

Based on the results of survival analyses and correlation studies, we tested whether the combination of markers had a higher predictive power than a single marker. As shown in Figure 4, the combination of high levels of* CD14*,* FOXP3*, and* ARG1* expression strongly predicted good EFS and OS. However, low levels of* CD14* remained the best predictor of a dismal survival (Figure 2(b)).

### 3.4. Analysis of a Public NB Tumor Gene Expression Profiling Dataset

**Figure 5:**
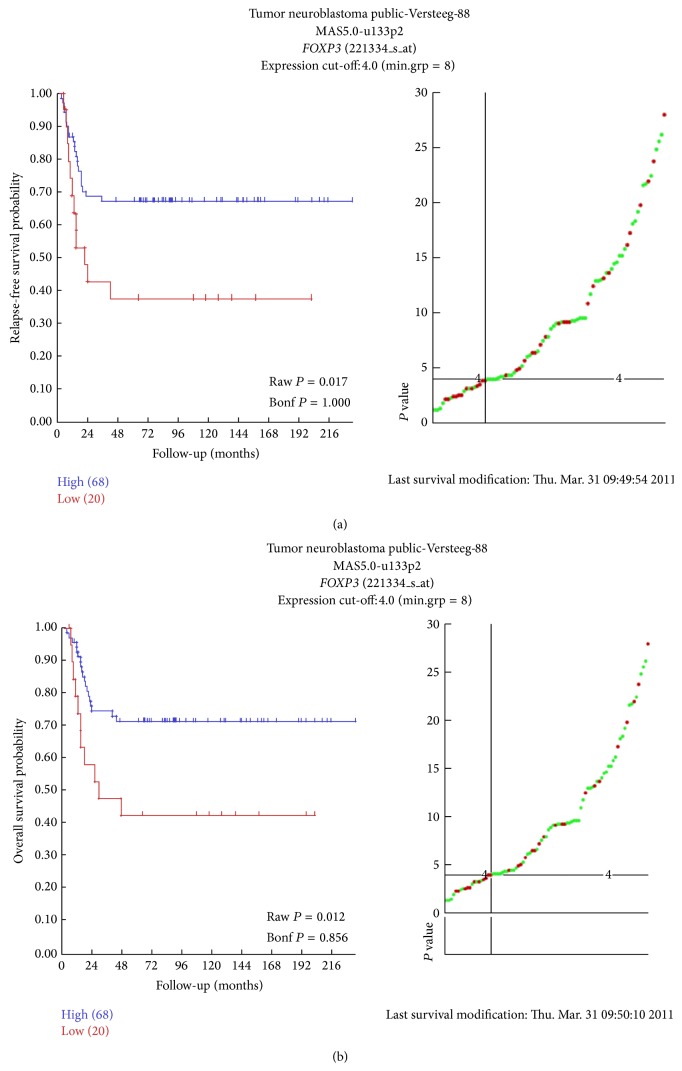
Kaplan-Meyer plots of EFS (a) and OS (b) of stage 4 patients from the public NB database stratified according to the level of* FOXP3* mRNA expression below or above the ROC determined cut-off.

We checked in a public gene expression profiling dataset of 40 stage 4 NB tumors (R2: Genomics Analysis and Visualization Platform: http://hgserver1.amc.nl/cgi-bin/r2/main.cgi, tumor neuroblastoma, Versteeg) significant associations between the expression levels of the studied molecular markers and EFS and OS. It is important to note that the public dataset reports microarray data and that Kaplan-Meyer plot is given for each probe according to ROC analysis. Despite the difference in the type of data and analysis, the significant association of high* FOXP3* mRNA levels with better EFS and OS was confirmed (Figure 5). No significant association was found for* CD14* and* ARG1* (not shown).

## 4. Discussion

The prognostic role of molecular analysis of immune cells and of the immunosuppressive cytokine* IL10* has been evaluated in primary tumors from 41 children with metastatic NB aged more than 18 months at diagnosis. The results indicate that high level of* CD14*,* ARG1*, and* FOXP3* mRNA expression in primary NB tumor significantly correlated to a better survival. Each of these markers had predictive power; however, the combined use of the three markers improved survival prediction and allowed to identify patients that can be cured by standard therapy. Furthermore, the association of* FOXP3* mRNA levels with different EFS and OS was confirmed in a gene expression dataset of 40 stage 4 NB tumors analyzed by microarray. The discrepancy in the predictive power of* CD14* and* ARG1* observed by RT-qPCR and microarray analysis may relate to the different sensitivity/specificity of the oligonucleotide probes used. Moreover, in the Versteeg's database the tumor cell content required for inclusion was lower and patients < 18 months could not be excluded. Thus, our findings need to be confirmed by qPCR in a prospective study with a greater cohort of stage 4 NB patients > 18 months.

The demonstration that* FOXP3* expression positively associated to a better EFS and OS of high-risk NB patients is in agreement with previous reports in head and neck and colorectal cancer patients [29, 30]. It has been suggested that activation state or suppressive functions of the infiltrating T cells greatly depend on the specific microenvironment present in the anatomical site [21].* FOXP3* expression did not correlate to* CD4* expression but correlated to* PFR1* mRNA levels, further supporting the tenet that* FOXP3* mRNA expression in NB tumors was an indicator of effector T-cell activation rather than of immunosuppressive Treg cells [21, 22, 31–33]. Since* FOXP3* expression correlated to* CD14* expression, the presence of macrophages or dendritic cells expressing* CD14* may be responsible for the activation of effector T cells, which in turn limited tumor progression. It is of note that* CD14* expression levels, but not those of the TAM marker* CD163*, significantly associated to different survival, suggesting that CD14+ cells other than TAMs may favor the induction of an effective immune response.

Regarding the role of FoxP3-expressing cells, in murine syngeneic NB models, CD4+FoxP3+ Treg cells increase in secondary lymphoid organs of NB-bearing mice and their depletion increased the effects of immunotherapy and of hematopoietic stem cell transplantation [34–37]. The apparent discrepancy between syngeneic murine models and human NB may relate to a different expression of FoxP3 in activated effector T cells in mouse and human [33]. In addition, human NB cells express low, if any, levels of HLA class I molecules [38]. This defect may hamper their recognition by CD8+ T cells but on the other hand may facilitate NK cell-mediated killing [39]. Nonetheless, HLA class I molecule expression can be restored in human NB cells by IFN-*γ* [40], produced by activated T and NK cells, allowing NB cell recognition by CTLs. It is important to note that NKT cells have been involved in the immune response to human NB [18] and that activated NKT cells may express FoxP3 [41]. Therefore a possible role of activated NKT cells cannot be excluded. Finally, since low expression of all tested molecular markers, although not significantly, associated to a worse survival, the possibility that all infiltrating immune cells may play a role in improving antitumor responses needs to be considered.

Taken together, our findings suggest that if the primary tumor of stage 4 NB patients >18 months is infiltrated by FoxP3-expressing effector T or NKT cells, an effective antitumor response may take place and cooperate with standard therapy to increase survival.

## 5. Conclusions

High expression of* FOXP3*,* CD14*, and* ARG1* mRNA in the primary tumors of high-risk NB patients was predictive of better survival, suggesting that the immune status of the tumor may influence the natural history of this pediatric cancer.

## Figures and Tables

**Table 1:** Patients' characteristics.

	*N*	%
Age at diagnosis		
<18 months	0	
>18 months	41	*100 *
Sex		
Female	17	*41.5 *
Male	24	*58.5 *
MYCN status		
Amplified	13	*31.7 *
Not amplified	28	*68.3 *
Primary tumor site		
Adrenal	25	*61.0 *
Thorax	3	*7.3 *
Abdomen	13	*31.7 *
Metastatic sites		
Bone Marrow	13	*31.7 *
Bone Marrow + bone	24	*58.6 *
Pleura	2	*4.9 *
Bone	1	*2.4 *
Other	1	*2.4 *
Relapse		
No	15	*36.6 *
Yes	26	*63.4 *
Outcome		
Alive	16	*39.0 *
Dead of disease	25	*61.0 *
